# Highly Reflective Thin-Film Optimization for Full-Angle Micro-LEDs

**DOI:** 10.1186/s11671-021-03611-1

**Published:** 2021-10-09

**Authors:** Zhi-Ting Ye, Wen-Tsung Ho, Chia-Hui Chen

**Affiliations:** 1 Department of Mechanical Engineering, Advanced Institute of Manufacturing with High-Tech Innovations, National Chung Cheng University, 168, University Rd., Min-Hsiung, Chia-Yi, 62102 Taiwan; 2 Department of R&D, General Manager’s Office, TO2M Corporation, Hsinchu, 30010 Taiwan

**Keywords:** Micro-LEDs, Highly reflective thin film, Secondary optical lens, Full-angle, Primary optical design

## Abstract

Displays composed of micro-light-emitting diodes (micro-LEDs) are regarded as promising next-generation self-luminous screens and have advantages such as high contrast, high brightness, and high color purity. The luminescence of such a display is similar to that of a Lambertian light source. However, owing to reduction in the light source area, traditional secondary optical lenses are not suitable for adjusting the light field types of micro-LEDs and cause problems that limit the application areas. This study presents the primary optical designs of dielectric and metal films to form highly reflective thin-film coatings with low absorption on the light-emitting surfaces of micro-LEDs to optimize light distribution and achieve full-angle utilization. Based on experimental results with the prototype, that have kept low voltage variation rates, low optical losses characteristics, and obtain the full width at half maximum (FWHM) of the light distribution is enhanced to 165° and while the center intensity is reduced to 63% of the original value. Hence, a full-angle micro-LEDs with a highly reflective thin-film coating are realized in this work. Full-angle micro-LEDs offer advantages when applied to commercial advertising displays or plane light source modules that require wide viewing angles.

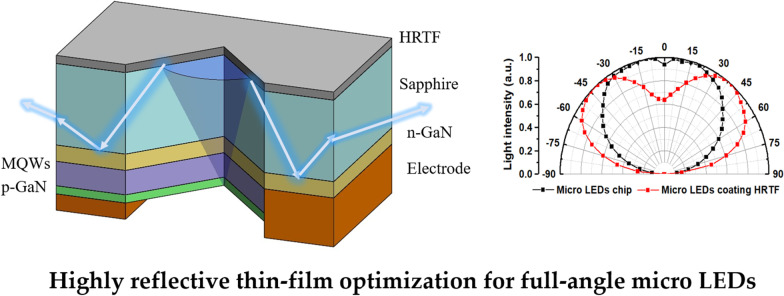

## Introduction

Displays have become an indispensable part of human life, including smartphones, computer monitors, television (TV), and commercial advertising screens, which are some examples of the most used display technologies. The current mainstream display technologies include liquid crystal displays (LCDs), organic light-emitting diodes (OLEDs), and micro-sized light-emitting diodes (micro-LEDs) [1–3]. LCDs have advantages such as long life, low price, and mature technology [4–6]; however, the overall light output efficiencies of large-sized direct-lit backlight LCDs are still low and their structure is complex, which makes it difficult to reduce the overall thickness [7–9].

OLEDs have the advantages of self-luminescence when applied to displays, small size, high flexibility, high contrast, and wide color gamut [10–12]; however, to solve the problem of poor color purity caused by mixing of the red, green, and blue sub-pixels when emitting light, it is necessary to use complex and fine metal masks, which limit the resolution and brightness of OLED displays as well as reduce their overall life spans owing to the characteristics of the internal organic materials [13–15].

Micro-LEDs have the advantages of high brightness, long life, and high efficiency, in addition to the advantages of LCDs and OLEDs [16–18]. Micro-LEDs displays are self-luminous and use extremely small micro-LEDs chips as point light sources, thereby offering advantages of high luminous efficiency, long life, high color purity, high contrast, and high chemical stability [19–21]; however, such displays still have challenges, such as shrinkage of the micro-LEDs sizes and relatively high substrate accuracy of the equipment, thereby causing problems with the transfer technology of a large number of micro-LEDs [22–24].

In addition to the difficulties with the manufacturing process, when using micro-LEDs as light sources, the displayed light field patterns have Lambertian characteristics, which causes problems such as limited viewing angles when applied to commercial advertising displays [25]. Thus, increasing the light-emitting angles of micro-LEDs not only increases the viewing angles of displays but also reduces their numbers and thickness when used as the backlights of LCDs. Thus far, there is still a lack of research on optimizing the light-emitting angles of micro-LEDs, so improving this area of study is expected to be beneficial [26–28]. In recent years, scholars have proposed optical designs to optimize the light-emitting angles. Spägele et al. proposed supercell metasurfaces (SCMS) that use the coupling between adjacent atoms in the supercell to achieve wide-angle effects; Estakhri et al. proposed the design of a highly efficient back-reflected visible light gradient metasurface composed of TiOx nanowires to achieve wide angles; Deng et al. proposed thin metal nano-gratings with rectangular grooves to construct metasurfaces to increase the light exit angles [29–31]. Qiu et al. proposed Au nanomesh structures with disordered double-sized apertures as a new type of transparent conductive film to achieve a wide viewing angles; Liu et al. proposed using graphene as a transparent conductive film because of its advantages of optical anisotropy and high light transmittance in large-angle incident areas; additionally, for infrared LEDs, Lee et al. studied the development of titanium–indium–tin oxide (TITO) thin films for low-temperature near-infrared light-emitting diodes (NIR-LEDs) by inserting 2-nm-thick Ti barriers between the top layers of the NIR-LEDs and ITO to achieve wide-angle effects [32–34].

Research related to modulating the light distributions using secondary optical elements have also been reported. Run et al. designed a new free-form surface lens whose inner surface is a cylinder and outer surface is a free-form surface to optimize the light-emitting angles; Lin et al. proposed a Cartesian candela-distributed free-form lens array to optimize the LED lens array layout to achieve wide angles [35, 36]. In addition, research on modulation of the light shape for Chip Scale Package-Light-emitting diode (CSP LEDs) include changing the traditional packaging structures and light distribution optimization for flat light sources [37, 38].

Several researchers have also considered various LED substrate designs to change the light field patterns. Lai et al. used a sulfuric acid wet etching process to form a triangular pyramid pattern on c-plane sapphire substrates to achieve higher light extraction efficiencies and increase the light angles; Lan et al. proposed a patterned sapphire substrate (PSS) combined with packaged inverted trapezoidal flip-chip micro-LEDs that show strong peaks and large light angles; Zhang et al. studied flip-chip deep-ultraviolet LEDs with nano-patterned sapphire substrate (NPSS) structures to show that the NPSS structure can achieve wide angles and enhance light extraction efficiency [39–41]. Optical components have also been added to optical modules to modulate the light distributions. Wang et al. proposed a compact high-directional backlight module combined with a striped diffuse reflector to diffuse light through a compact light guide plate and realize wide viewing angles; Li et al. designed a quarter-wave plate of a multi-twist retarder to achieve achromatic aberration effects and wide viewing angles [42, 43].

To achieve a wide viewing angle, the LCD must be design and match wide-angle backlit and liquid crystal material. In this process, there are problems of lateral light leakage and color shift. With three groups directional backlights and a fast-switching LCD panel, a time-multiplexed light field display with a 120-degree wide viewing angle is demonstrated [44].

Thus, previous research on improving the light-emitting angles lack relevant investigations into the design of optical films on micro-LEDs chips to increase the light-emitting angles. As the sizes of micro-LEDs have been greatly reduced in recent times, it is impossible to adjust the light field types using secondary optical lenses as in traditional LEDs. Previous studies have also proposed adjusting the light field types with metal films; metals have excellent reflectivity at different angles, but the materials have high light absorption coefficients that reduce the light output efficiencies. The reflectivity of dielectric materials at different angles is not relatively better than those of metals, but the materials themselves have low light absorption coefficients. This paper proposes a primary optical design for dielectric and metal films to obtain low-absorption and high-reflectivity thin films deposited on the surfaces of micro-LEDs and achieve full-angle light distribution while accounting for the light output efficiencies and full-angle light emissions of the micro-LEDs. Full-angle micro-LEDs offer advantages when applied to commercial advertising displays or plane light source modules that require wide viewing angles.

## Materials and Methods

### Micro-LEDs Chip Sizes and Light Field Types

**Fig. 1:**
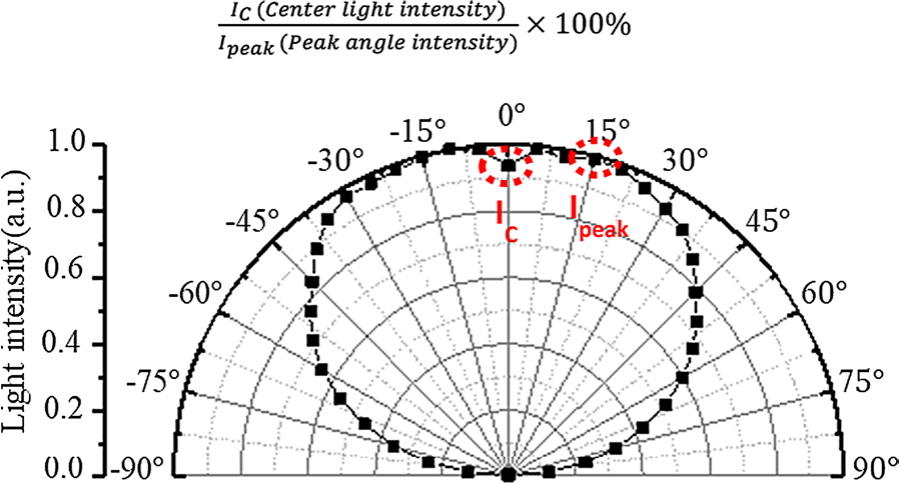
Micro-LEDs chip light distribution curve

The dimensions of the micro-LEDs used in this study based on length *L*_c_, width *W*_c_, and height *H*_c_ are 150 µm, 85 µm, and 85 µm, respectively. The light distribution curve of the bare chip is shown in Fig. 1. The intensity of the center point in the normal direction *I*_C_ is 92%, the peak angle *I*_peak_ is 15°, and the calculation method for the intensity of the center point is expressed by Eq. (1). From the light distribution curve, it is seen that micro-LEDs have similar Lambertian light types, with a full width at half maximum (FWHM) of 135°; therefore, increasing the light-emitting angles to obtain full-angle luminescence without the secondary optical lens is the main focus of research in this work.

1 $$\frac{{I_{{{\text{C}} }} \,\left( {{\text{Center}}\,{\text{light}}\,{\text{intensity}}} \right)}}{{I_{{{\text{peak}}}} \,\left( {{\text{Peak}}\,{\text{angle}}\,{\text{intensity}}} \right)}} \times 100\%$$

**Fig. 2:**
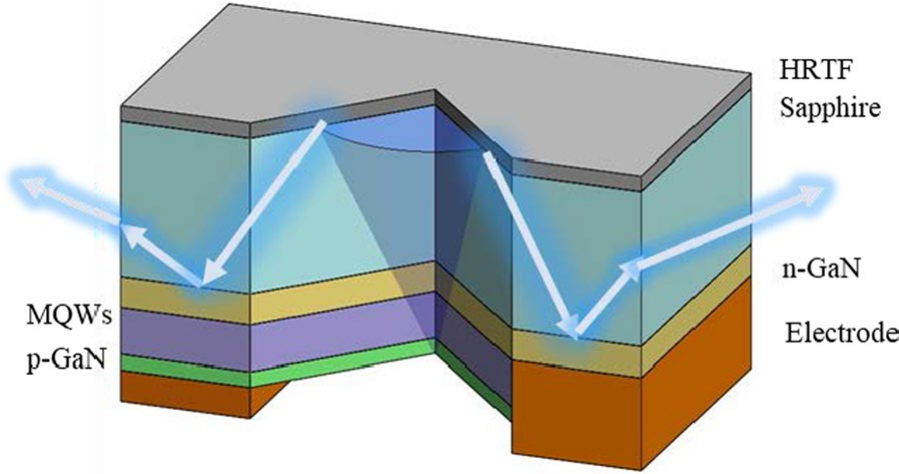
The light path within the full-angle micro-LEDs with HRTF coating

Among the aforementioned parameters, low central light intensity and increased peak luminous angle help improve the uniformity and viewing angle [45]. This study presents the design of a highly reflective thin-film (HRTF) layer on the surface of the micro-LEDs chip, which includes a dielectric film made of TiO_2_/SiO_2_ stacked dielectric materials and a metal film made of Al. The structure of the micro-LEDs and the light path through it are shown in Fig. 2. The light exits through the multiple quantum wells (MQWs) layer and is partially reflected by the HRTF. Thereafter, the light exits from the sidewall of the Al_2_O_3_ layer, with an increased light exit angle from the micro-LEDs to realize a full-angle light exit.

### Materials of the HRTF

The choice of materials used in the optical film is crucial to achieve the desired characteristics. First, the material must have a low extinction coefficient in the required wavelength band to avoid reducing the light extraction efficiency owing to large absorption; then, the material’s adhesion, physical and chemical stabilities, and light transmittance must be considered. The dielectric material TiO_2_/SiO_2_ has excellent characteristics for these properties in the visible light band. Al has a relatively high extinction coefficient, but its reflectivity cannot be easily decreased with increasing incident angles; however, it can withstand high light intensities. Based on the above characteristics, the high refractive index material (*H*) TiO_2_ and low refractive index material (*L*) SiO_2_ are used for the dielectric film, and Al is used for the metal film, with Al_2_O_3_ as the substrate for the optical thin-film design. The refractive indices of the materials used in this study are shown in Table 1 at the dominant wavelength of 460 nm.

**Table 1:** Refractive indices and extinction coefficients of the materials used in this study at a dominant wavelength of 460 nm

Material	Refractive index at 460 nm
Al_2_O_3_	1.67026
TiO_2_	2.40695
SiO_2_	1.46488
Al	0.54095

### HRTF Design Optimization

The substrate used for the light-emitting surface of the micro-LEDs is Al_2_O_3_. We designed the HRTF on the substrate and used the dielectric and metal films to improve reflectivity while maintaining high luminous efficiency. The goal here was to achieve a reflectance > 90% at the dominant wavelength of 460 nm. The principle behind the design of the HRTF is to use the destructive and constructive interference characteristics of light to improve reflectivity. Maximum light interference in the film medium occurs when the optical thickness is 1/4 of the wavelength, and the interface reflectivity R at this time is calculated according to Eq. (2) [46].

2 $$R = \frac{{n_{{\text{s}}} n_{2}^{2P} - n_{{{\text{air}}}} n_{1}^{2P} }}{{n_{{\text{s}}} n_{2}^{2P} + n_{{{\text{air}}}} n_{1}^{2P} }}$$

Here, *P* is the number of TiO_2_–SiO_2_ periods,${ }n_{{\text{s}}}$ is the refractive index of the substrate, $n_{1}$ is the refractive index of TiO_2_, $n_{2}$ is the refractive index of SiO_2_, and $n_{{{\text{air}}}}$ is the refractive index of the air medium. The transmission optical thickness is 1/4 of the wavelength; hence, the physical thicknesses of Al, TiO_2_, and SiO_2_ are 20 nm, 47.78 nm, and 78.50 nm, respectively. This study uses the Macleod optical simulation software to simulate four thin-film structures for pure Al, Al/(HL), Al/(HL)^2^, and Al/(HL)^3^.

**Fig. 3:**
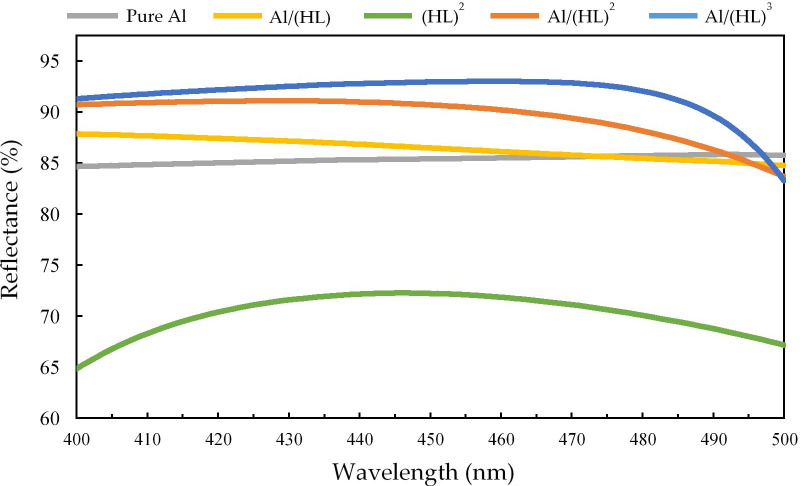
Reflectance of pure Al, Al/(HL), (HL)^2^, Al/(HL)^2^, and Al/(HL)^3^ was simulated at wavelengths of 400–500 nm

Figure 3 shows the relationship between the wavelength and reflectance of pure Al, Al/(HL), (HL)^2^, Al/(HL)^2^, and Al/(HL)^3^ of the five membrane stack structures in the simulated wavelength range of 400–500 nm. The reflectivity of pure Al, Al/(HL), (HL)^2^, Al/(HL)^2^, and Al/(HL)^3^ at 460 nm is 85.53%, 86.15%, 71.84%, 90.23%, and 93.04%, respectively.

Table 2 shows the reflectance, transmittance, and absorption ratios of the five kinds of membrane stack structures, namely pure Al, Al/(HL), (HL)^2^, Al/(HL)^2^, and Al/(HL)^3^. The transmittance rate of pure aluminum at 460 nm is 5% and absorption rate is 9.47%, which is the highest absorption rate among the five types of membrane stacks. The transmittance of the (HL)^2^ membrane stack at 460 nm is 28.06% and absorption rate is 0.1%; this absorption rate directly affects the overall light extraction efficiency; further, this membrane stack structure has the smallest absorption rate, and its reflectivity is only 71.84%. The Al/(HL)^2^ membrane stack has a transmittance of 4.38% at 460 nm and an absorption rate of 5.39%; this membrane stack structure takes into account the overall light extraction efficiency and full-angle light distribution. Considering both the radiant flux and overall light extraction efficiency, the Al/(HL)^2^ membrane stack structure was used in this study for the HRTF coating.

**Table 2:** Reflectance, transmittance, and absorption rates of pure Al, Al/(HL), (HL)^2^, Al/(HL)^2^, and Al/(HL)^3^ at 460 nm

	Transmittance (%)	Reflectance (%)	Absorption (%)
Pure Al	5	85.53	9.47
Al/(HL)	6.81	86.15	7.04
(HL)^2^	28.06	71.84	0.1
Al/(HL)^2^	4.38	90.23	5.39
Al/(HL)^3^	1.88	93.04	5.08

**Fig. 4:**
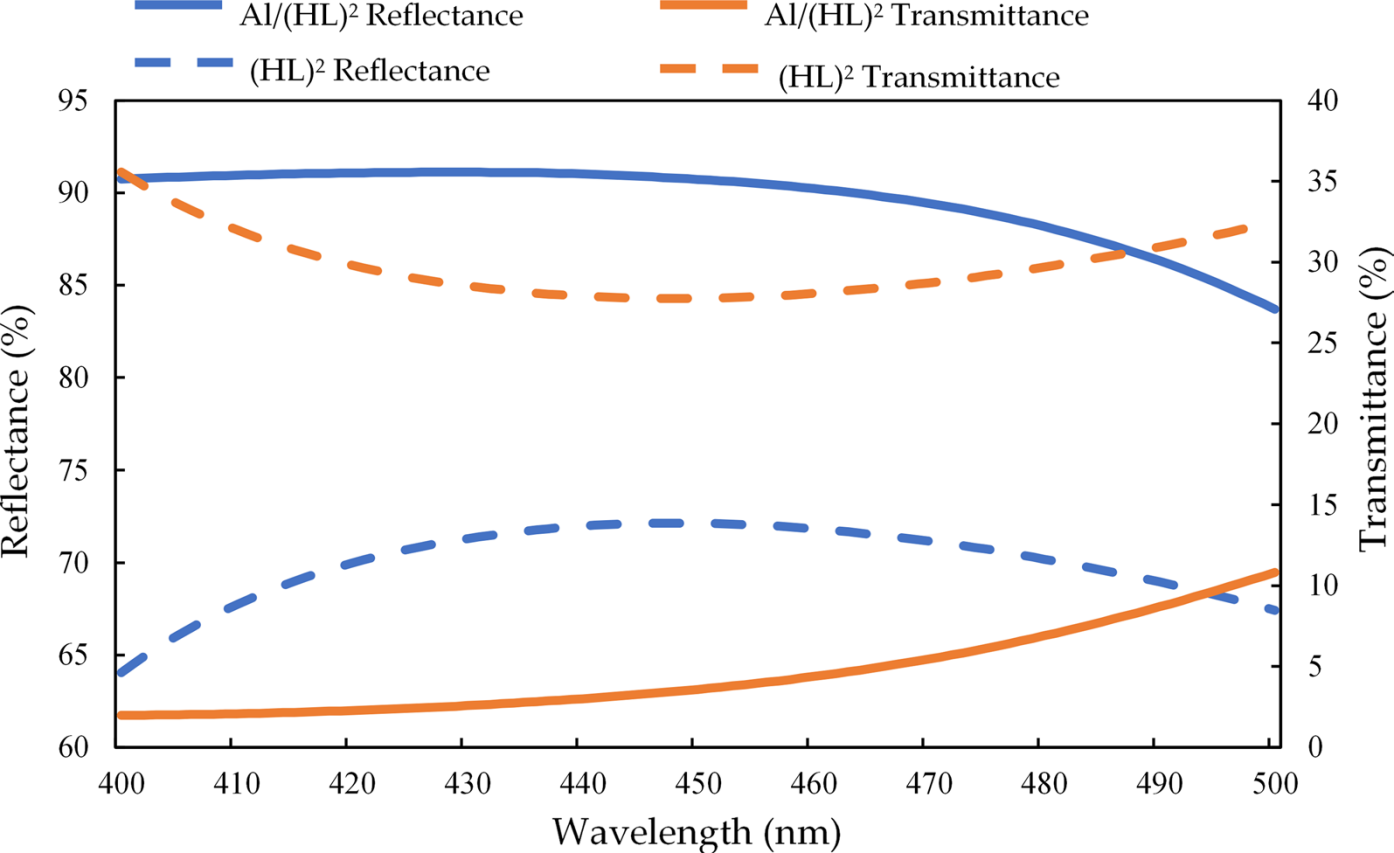
Reflectance and transmittance ratios of the simulated thin-film structures of Al/(HL)^2^ and (HL)^2^ for wavelengths in the range of 400–500 nm

Figure 4 shows the simulated Al/(HL)^2^ and (HL)^2^ as well as their corresponding reflectance and transmittance graphs for 400–500 nm. The average reflectance and transmittance of Al/(HL)^2^ are 89.6% and 4.54%, and the average reflectance and transmittance of (HL)^2^ are 70.3% and 29.56%, respectively. It can be seen from the simulation results that adding the thin aluminum layer increases the reflectivity by a factor of 1.27.

**Fig. 5:**
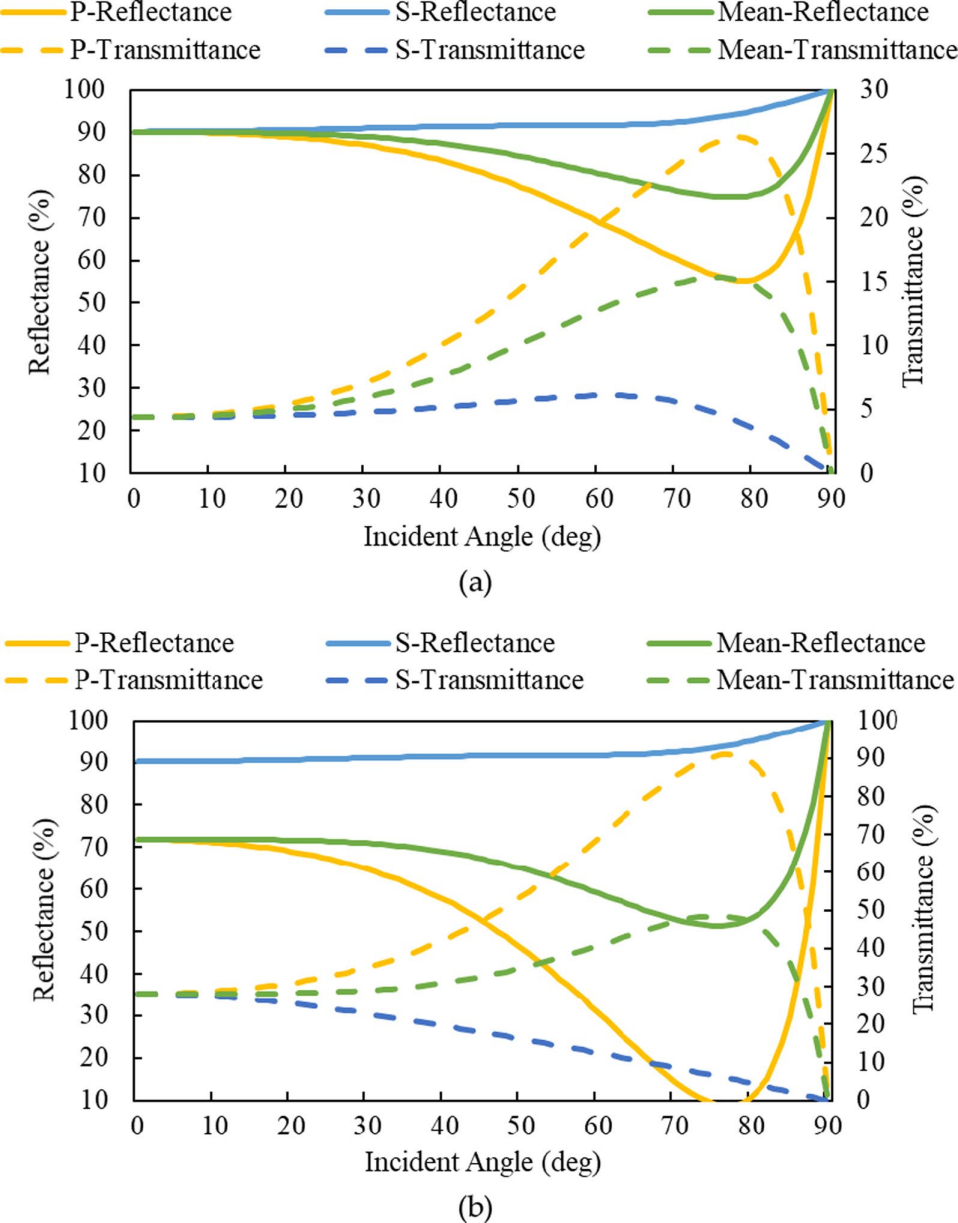
Reflectance and transmittance ratio changes of the simulated **a** Al/(HL)^2^ for incident angles of 0–90°and **b** (HL)^2^ for incident angles of 0–90°

Figure 5 illustrates the changes in (a) the transmittance and reflectance of Al/(HL)^2^ at different incident angles; from 0° to 60°, the average reflectance is 87.7% and average transmittance is 6.97%. Figure 5b. The transmittance and reflectance of (HL)^2^ at different incident angles; from 0° to 60°, the average reflectance is 68.99% and average transmittance is 30.88%. In full-angle reflective film design, Al/(HL)^2^ can be seen from the simulation results that adding the thin aluminum layer increases the full angle of average reflectance by a factor of 1.27.

**Fig. 6.**
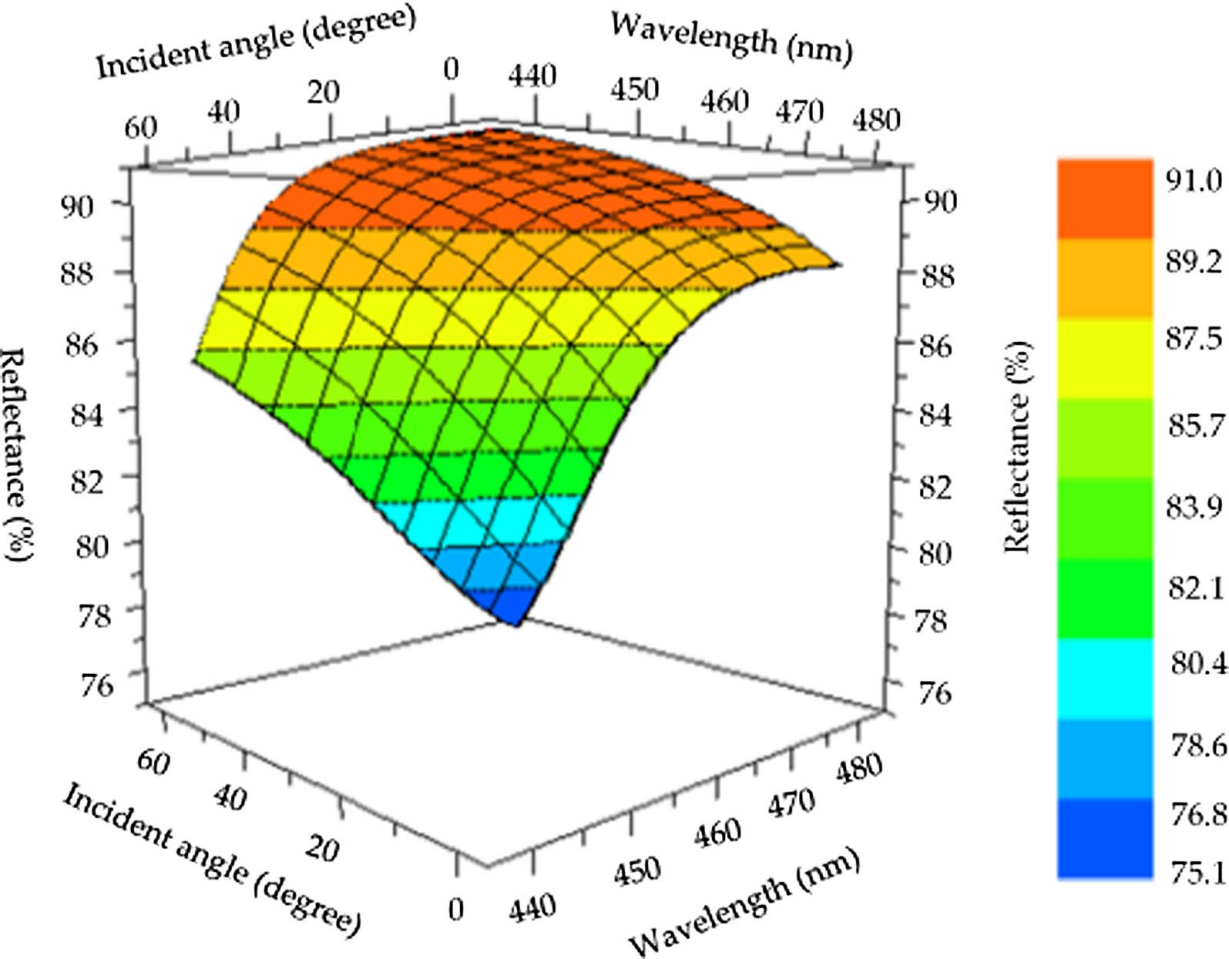
3D relationship diagram of the simulated wavelengths, incident angles, and reflectivity of Al/(HL)^2^

Figure 6 shows the simulated wavelength/incidence angle/reflectivity 3D diagram of Al/(HL)^2^ for incident angles of 0–25° and average reflectivity exceeding 90% in the wavelength range of 440–480 nm.

## Results and Discussion

**Fig. 7:**
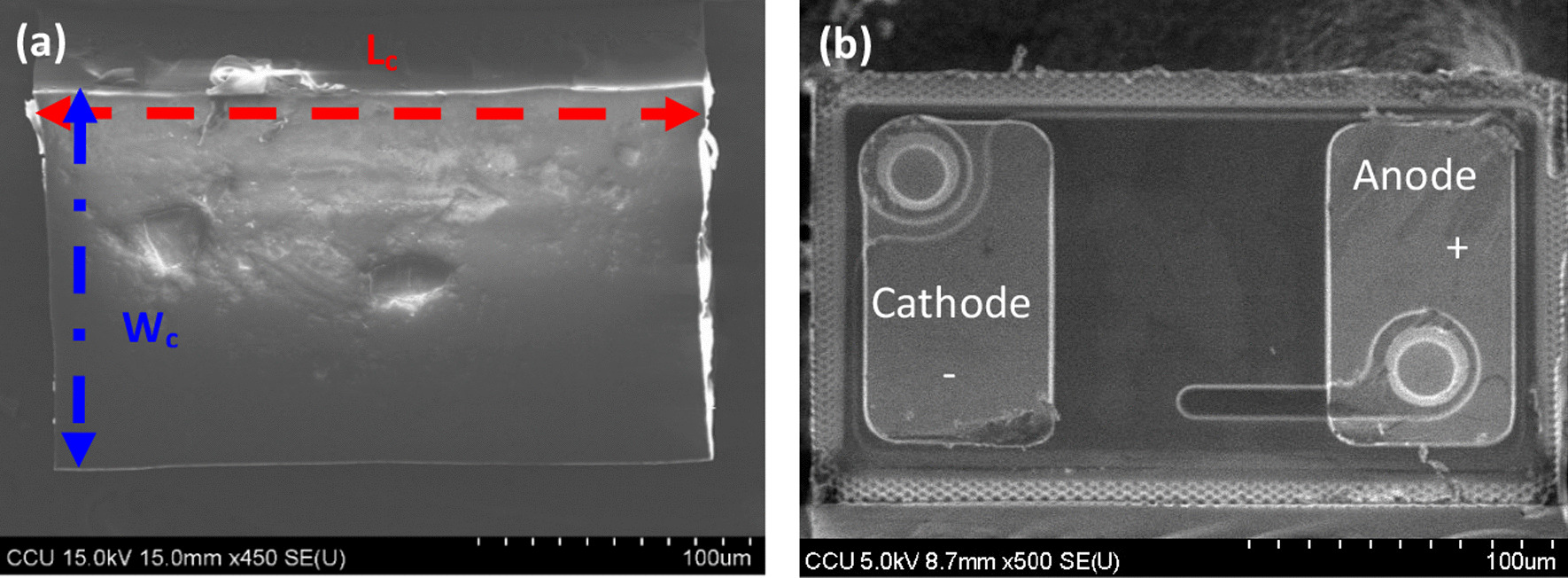
SEM images of micro-LEDs chip: **a** top and **b** bottom views

**Fig. 8:**
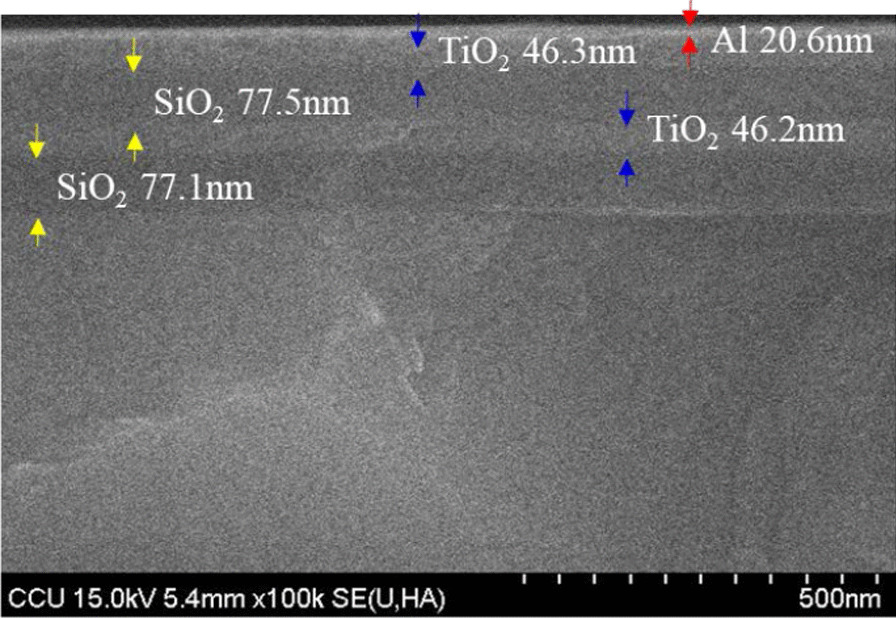
Cross-sectional SEM image of the HRTF

Figure 7 shows the scanning electron microscope (SEM) images of the HRTF coating of the micro-LEDs chip. The chip length L_c_ is 240 µm, width W_c_ is 140 µm, and height H_c_ is 100 µm. Figure 8a shows the top view, and Fig. 8b shows the bottom view.

Figure 8 shows the cross-sectional SEM image of the micro-LEDs chip with HRTF coating. The HRTF prototype film stack includes an Al film thickness of 20.6 nm, TiO_2_ dielectric film thicknesses of 46.3 nm and 46.2 nm, and SiO_2_ dielectric film thicknesses of 77.5 nm and 77.1 nm.

**Fig. 9:**
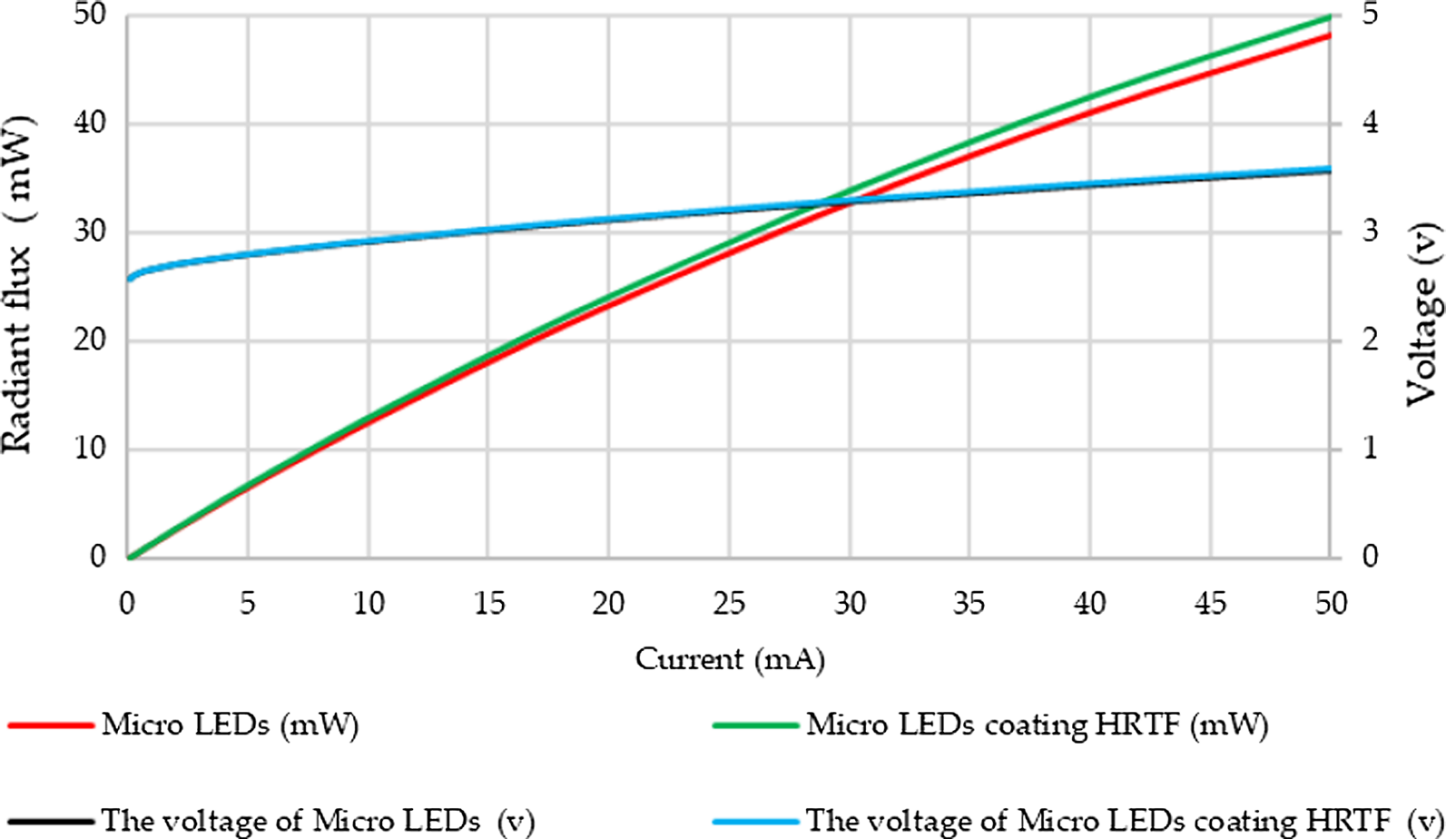
Photoelectric characteristics of the micro-LEDs without and with HRTF coating

Figure 9 shows the measured luminance–current–voltage (L–I–V) curve. Under an input current of 30 mA, the results show that without the HRTF coating, the output radiation flux, the voltage, and external quantum efficiency (EQE) are 33.833 mW, 3.293 V, and 41.84%, respectively. The voltage, output power, and EQE of the HRTF coating are 3.301 V, 32.757 mW, and 40.51%, respectively. The results show the HRTF coating hardly affects the current versus voltage (IV) curve characteristics of the micro-LEDs. The EQE of HRTF coating is decay 3.178%.

As the input current increases to 50 mA, this voltage and output power increase to 3.5 V and 48.165 mW, respectively, and the radiant flux is only about 3.3% lower than that of the micro-LEDs without the HRTF coating. This shows that micro-LEDs with HRTF coatings have low voltage variation rates and low optical losses characteristics.

**Fig. 10:**
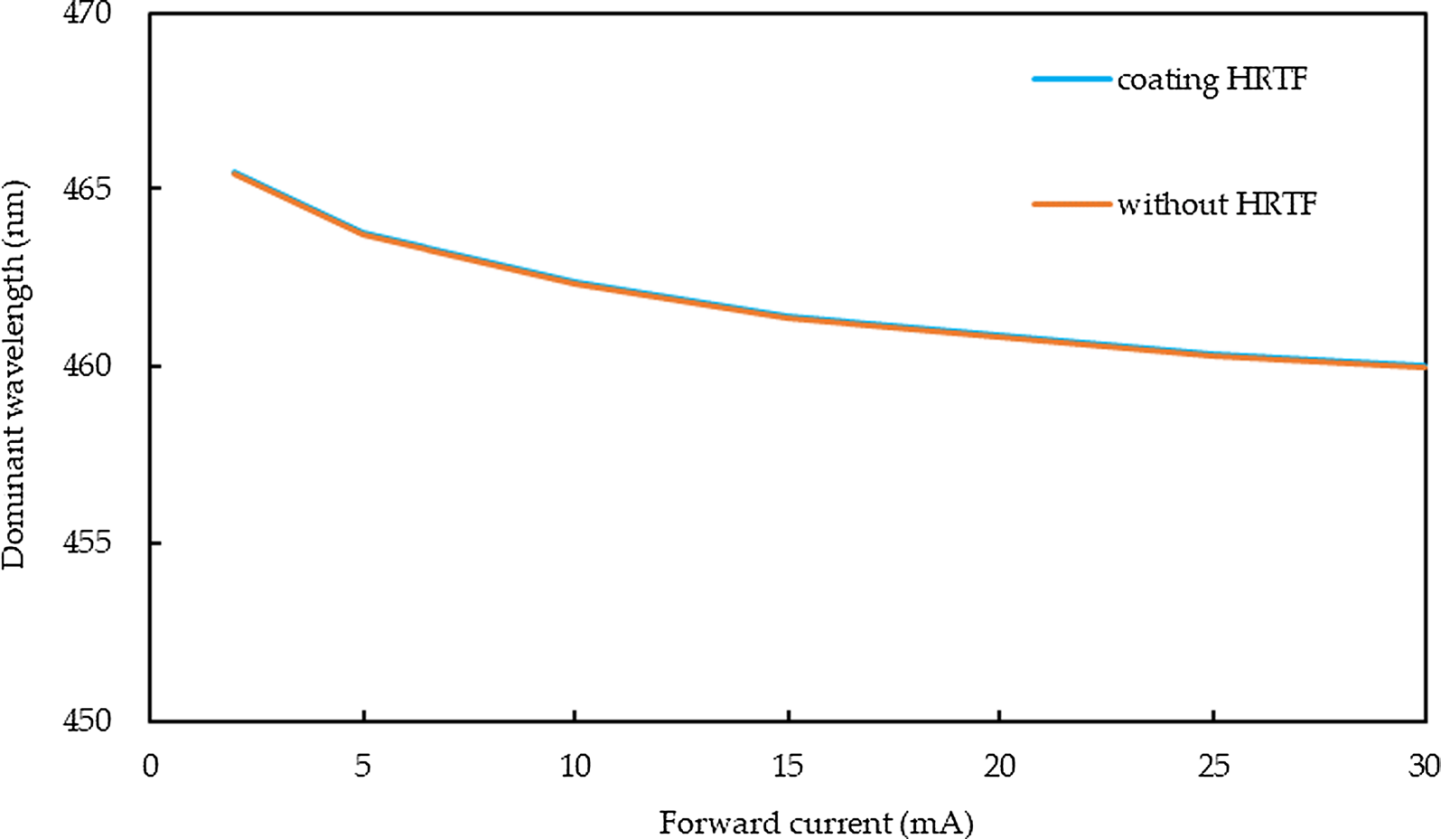
Changes in the dominant wavelength characteristic curves of micro-LEDs with and without Al/(HL)^2^ film stack coating

Figure 10 shows the drift characteristics of the dominant wavelength of the current for the micro-LEDs with HRTF stack coatings. The orange line represents the bare micro-LEDs and blue line is the micro-LEDs with HRTF coating. When the current increases from 2 to 30 mA, the peak wavelength changes from 465.47 to 460.01 nm, indicating that the micro-LEDs coated with the stack of Al/(HL)^2^ membranes show only 5.46 nm change for the dominant wavelength of the current; hence, these results show that the photoelectric properties of the original bare micro-LEDs are maintained.

**Fig. 11:**
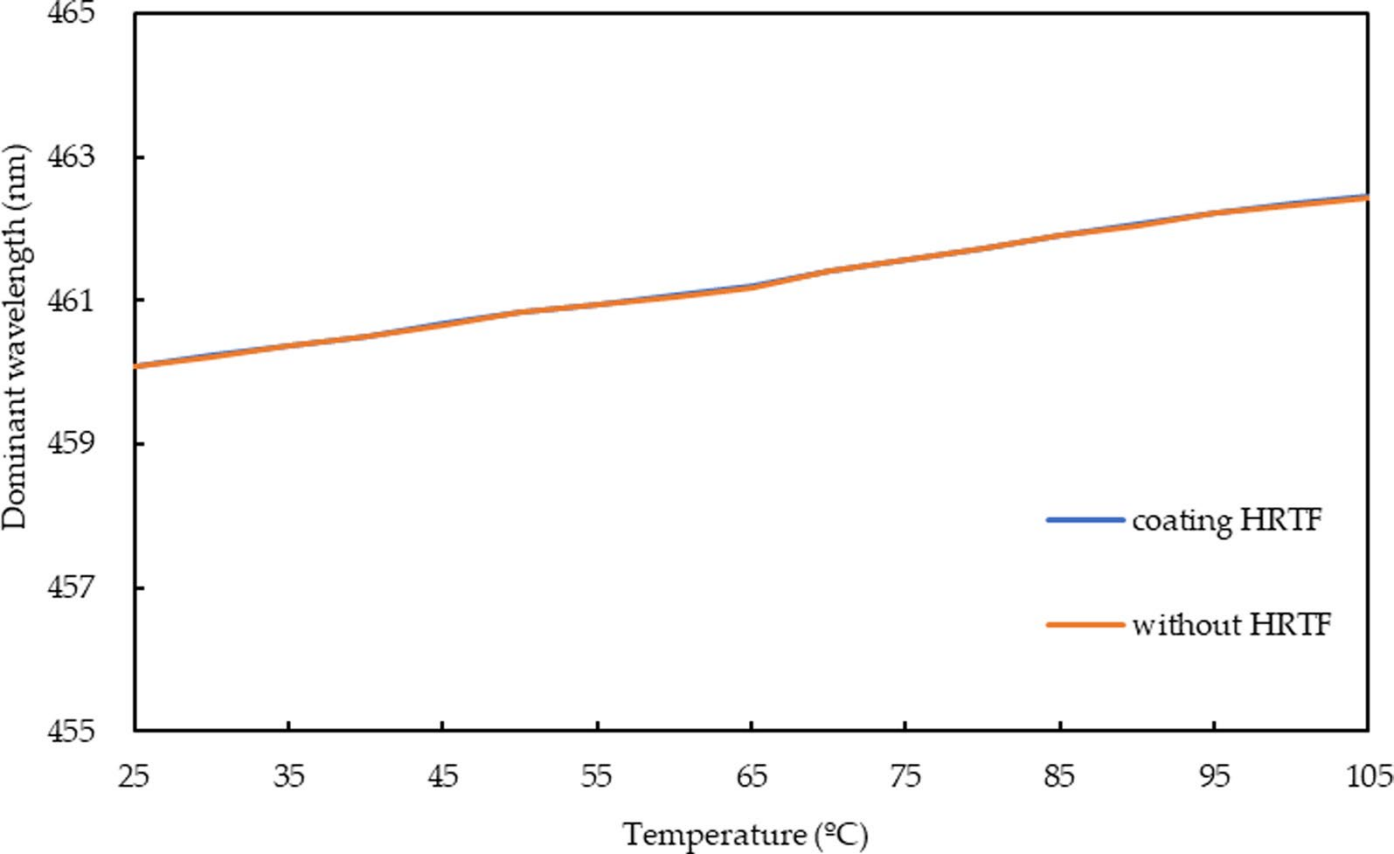
Characteristic curves of the peak wavelengths for micro-LEDs with and without Al/(HL)^2^ film stack coatings based on temperature variations

Figure 11 shows the temperature versus peak wavelength characteristic curves. The orange line represents the bare micro-LEDs, and the blue line is the micro-LEDs with HRTF coating. As the temperature increases from 25 to 105 °C, the peak wavelength is red-shifted from 460.09 to 462.45 nm; these two curves show that the original photoelectric characteristics are still maintained after the HRTF coating. The dominant wavelength shift is only 2.36 nm.

**Fig. 12:**
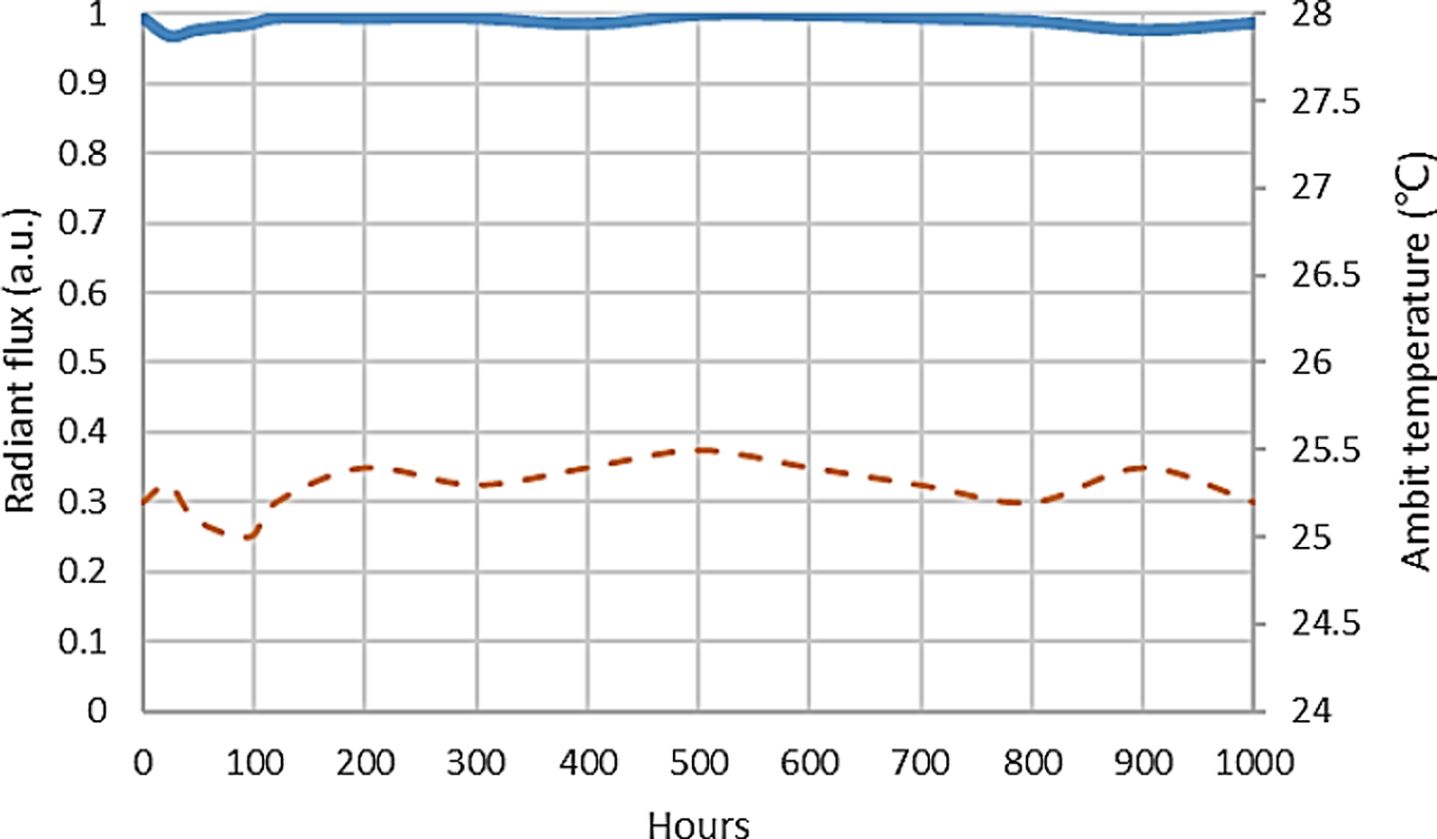
The long-term stability test of HRTF

The long-term stability test of HRTF is shown in Fig. 12. The test ambit temperature is 25 ℃ and the drive current is 30 mA. At 1000 h, the radiant flux can be maintained at 98.5%.

**Fig. 13:**
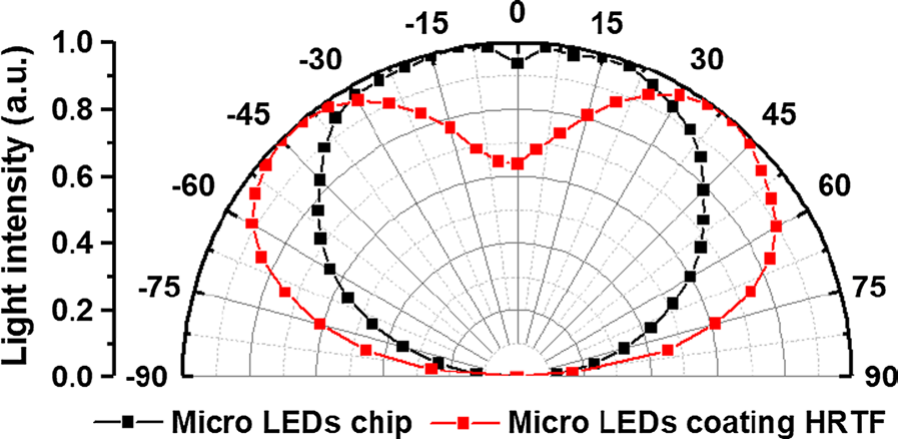
Light distribution curves of bare and HRTF-coated micro-LEDs

Figure 13 shows the light distribution curves of the bare and HRTF-coated micro-LEDs. The black line represents the light field pattern of the bare micro-LEDs, whose FWHM is 135°, center light intensity is 92%, and peak angle is 15°. The red line represents the light distribution of the micro-LEDs with HRTF coating, whose FWHM is increased to 165°, center light intensity is reduced to 63%, and peak angle is increased to 37.5°.

**Fig. 14:**
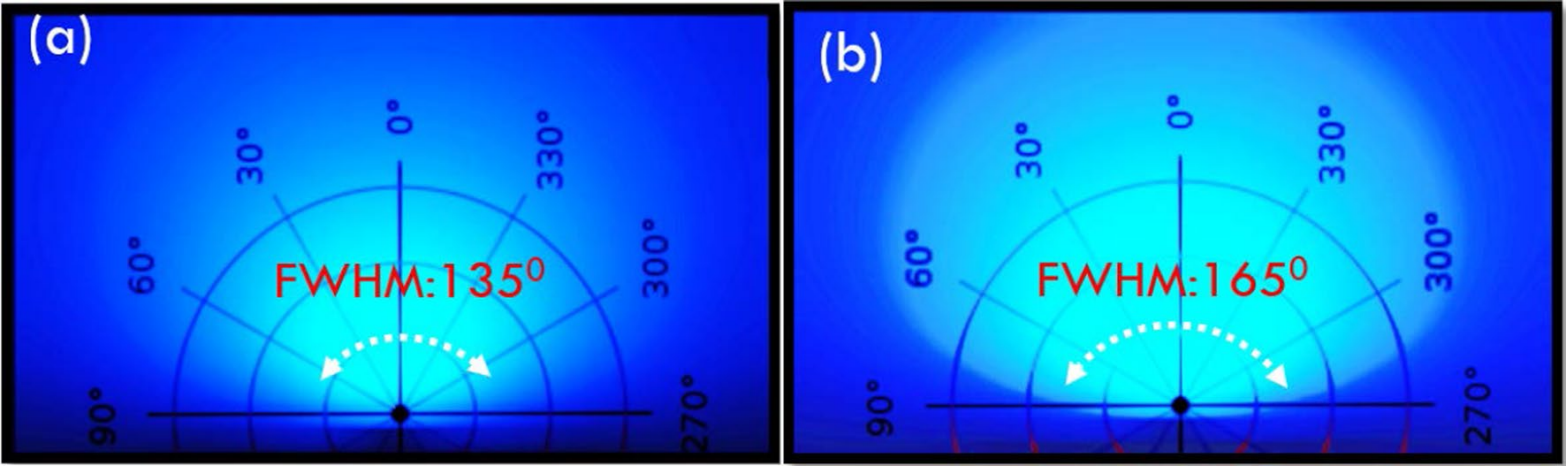
Schematic of the luminous distributions of **a** bare and **b** HRTF-coated micro-LEDs

Figure 14 shows the diagram of the luminous distributions of the (a) bare and HRTF-coated micro-LEDs. Figure 14b shows that the luminous distribution of the micro-LEDs with HRTF coating has wider angles and a more uniform distribution.

**Fig. 15:**
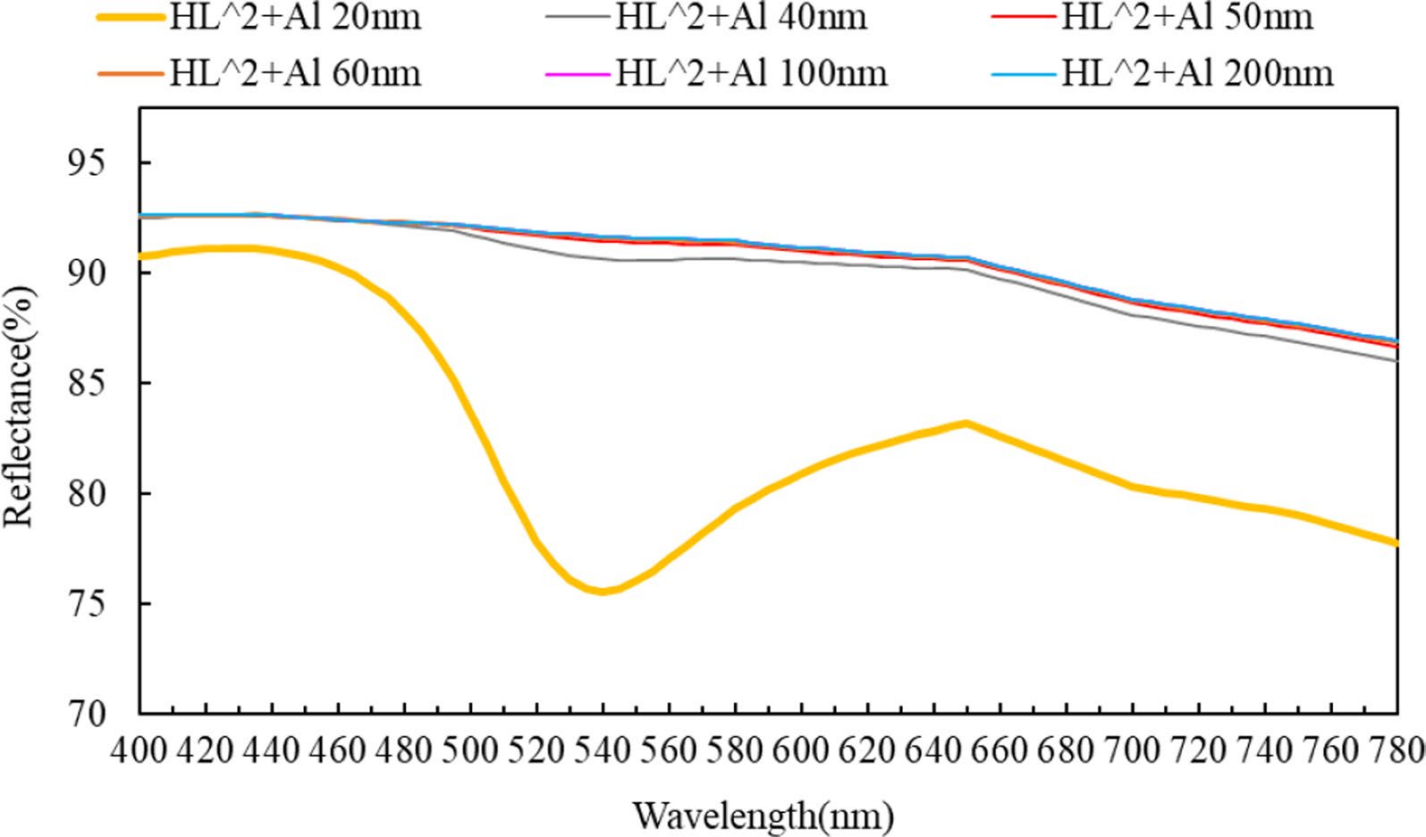
Reflectance relationship of different wavelengths corresponding to HRTF

The chromatic aberration between the different areas of the HRTF as a large wide-angle display screen is shown in Fig. 15.

This article is based on the wavelength range of 440–460 nm to optimize the design of HRTF. If it is applied to full color in the future, the thickness of the aluminum film be increased to 50 nm or more, and it will be better color uniformity at the global wavelength (400–780 nm).

## Conclusions

We propose the design of a HRTF coating on the surfaces of micro-LEDs to increase their light distribution angles to achieve full viewing angles. We use a primary optical design to modulate the light shapes of the micro-LEDs without secondary optical elements. The HRTF film stack structure is optimized using Al/(HL)^2^ to obtain high reflection and low absorption. Measurements on prototype fabricated micro-LEDs show that the L–I–V curve has almost no impact on the I–V characteristics of the micro-LEDs under an input current of 30 mA with the HRTF coating, and the radiation flux is only 3.3% lower than that of the bare micro-LEDs. In terms of light-emitting angles, the center light intensities of the micro-LEDs with HRTF coating are reduced from 92 to 63%, the peak angle increases from 15° to 37.5°, and the FWHM is enhanced from 135° to 165°.

The results of evaluation experiments show that micro-LEDs with HRTF coating have low voltage variation rates, low optical losses, and large full-angle light distribution of 165°. The full-angle micro-LEDs are fabricated with consideration of the overall light efficiency while still maintaining the photoelectric characteristics of bare micro-LEDs; these micro-LEDs offer advantages when applied to displays or plane light source modules that require wide viewing angles.

## Data Availability

The datasets supporting the conclusions of this article are available in the article.

## Abbreviations

micro-LEDs: Micro-light-emitting diodes
FWHM: Full width at half maximum
TV: Television
LCDs: Liquid crystal displays
OLEDs: Organic light-emitting diodes
SCMS: Supercell metasurfaces
TITO: Titanium–indium–tin oxide
NIR-LEDs: Near-infrared light-emitting diodes
CSP-LEDs: Chip Scale Package-Light-emitting diode
PSS: Patterned sapphire substrate
NPSS: Nano-patterned sapphire substrate
*L*_c_: Micro-LEDs length
*W*_c_: Micro-LEDs width
*H*_c_: Micro-LEDs height
*I*_peak_: Peak angle intensity
*I*_C_: Center light intensity
HRTF: Highly reflective thin film
MQW: Multiple quantum well
*H*: High refractive index material
*L*: Low refractive index material
*k*: Extinction coefficient
SEM: Scanning electron microscope
L–I–V: Luminance–current–voltage
IV: Current versus voltage
